# The circRNA interactome–innovative hallmarks of the intra- and extracellular radiation response

**DOI:** 10.18632/oncotarget.19228

**Published:** 2017-07-13

**Authors:** Valerie Bríd O'Leary, Jan Smida, Martina Matjanovski, Corinna Brockhaus, Klaudia Winkler, Simone Moertl, Saak Victor Ovsepian, Michael John Atkinson

**Affiliations:** ^1^ Institute of Radiation Biology, Helmholtz Zentrum Munich - German Research Center for Environmental Health, Neuherberg, Germany; ^2^ Institute of Biological and Medical Imaging, Helmholtz Zentrum Munich - German Research Center for Environmental Health, Neuherberg, Germany; ^3^ Faculty for Electrical Engineering and Information Technology, Technical University Munich, Munich, Germany; ^4^ Chair of Radiation Biology, Technical University Munich, Munich, Germany

**Keywords:** circRNA, WWOX, QUAKING, KIRKOS, exosomes

## Abstract

Generated by Quaking (QKI), circular RNAs (circRNAs) are newly recognised non-coding RNA (ncRNA) members characterised by tissue specificity, increased stability and enrichment within exosomes. Studies have shown that ionizing radiation (IR) can influence ncRNA transcription. However, it is unknown whether circRNAs or indeed QKI are regulated by IR. Microarray circRNA profiling and next generation sequencing revealed that circRNA expression was altered by low and medium dose exposure sourced predominantly from genes influencing the p53 pathway. CircRNAs *KIRKOS-71* and *KIRKOS-73* transcribed from the *WWOX* (*WW Domain Containing Oxidoreductase*) tumor suppressor (a p53 regulator) responded within hours to IR. *KIRKOS-71* and *KIRKOS-73* were present in exosomes yet exhibited differential transcript clearance between irradiated cell lines. Dual-quasar labelled probes and *in-situ* hybridization demonstrated the intercellular distribution of *KIRKOS-71* and *KIRKOS-73* predominantly within the perinucleus. QKI knockdown removed nuclear expression of these circRNAs with no significant effect on cytosolic *KIRKOS-71* and *KIRKOS-73.* Distinct QKI transcription between cell lines and its augmented interaction with *KIRKOS-71* and *KIRKOS-73* was noted post IR. This foremost study provides evidence that QKI and circRNAs partake in the cellular irradiation response. *KIRKOS-71* and *KIRKOS-73* as stable secreted circRNAs may afford vital characteristics worth syphoning as promising diagnostic radiotherapy biomarkers.

## INTRODUCTION

An awareness for circular RNA (circRNA) in the non-coding transcriptome came to light with elimination of selection bias only for polyadenylated RNA (poly A^+^) in high-throughput sequencing approaches [1]. Inclusion of bioinformatic criteria for scrambled exons enabled recognition of circRNA abundance and conservation in the mammalian transcriptome [2]. Representing covalently closed single stranded loops of ∼ 100 nucleotides [3] that lack polarities and poly A+ tails [4], circRNA were considered to be merely aberrant splicing by-products until recently. Furthermore, their stability and specific expression across developmental stages [3] pointed towards the hidden relevance of circRNAs in cellular homeostasis [1, 4, 5]. It has now become evident that circRNAs are conserved, exhibit tissue-specific expression patterns and are regulated independently from their linear counterparts [1, 2].

The first and most extensively characterised circRNA, CDR1as/ciRS-7 (Cerebellar degeneration-related protein 1 antisense), was demonstrated to suppress miR-7 with implications for cancer-related signalling pathways [6, 7]. Findings with a testis-specific circRNA, *Sry*, (Sex-determining region Y), purported to act similarly as a miR-138 sponge [8]. Comparative RNA-seq analysis of normal colon mucosa and colorectal tumours revealed circRNA reduction in malignant tissues [9], highlighting their potential as biomarkers or therapeutic targets [4, 10]. Moreover, circRNAs have already been shown to serve as biomarkers for the non-invasive diagnosis of atherosclerosis [11], central neural [12] and degenerative diseases [13]. CircRNAs/‘exo-circRNAs’ are at least two fold enriched in human exosomes [14, 15]. The exceptionally high stability of such circulating circRNAs has been attributed to the protection provided by exosomes, their specific sequence features or protein binding partners [14].

Quaking (QKI) binds intronic sites flanking exons to promote circRNA biogenesis [16]. Influencing pre-mRNA splicing and mRNA turnover [16], QKI (isoform 5) is predominantly located in the nucleus [17] and is directly regulated by the tumor suppressor p53 [5] a significant player in the cellular reaction to environmental stress *eg.* radiation [18]. The tumor suppressor WWOX, has been shown to be actively involved in the interaction with p53 to control genotoxic induced cell death [19]. We have found *WWOX* to be responsive to gamma irradiation (manuscript under consideration) and to produce a variety of circRNAs. The recent identification of the involvement of lncRNA [20] and miRNA [21] in the radiation response has turned the focus on whether *WWOX* circRNA expression might likewise be affected. This study hereby introduces two *WWOX* circRNAs *KIRKOS-71* and *KIRKOS-73*. We present evidence that upon irradiation exposure these circRNAs are independently controlled by QKI and ultimately destined for extracellular exosomal clearance.

## RESULTS

### Microarray screening and next generation sequencing reveal differential circRNA expression post irradiation

Datasets were sourced from RNA extracted 4 and 24 hr post low, medium dose or sham irradiated endothelial HUVEC cells. CircRNA differential expression was assessed initially from a human circRNA microarray (6 × 7K, Arraystar). A total of 3041 circRNAs were identified with 65.3 % (1986/3041) differentially expressed between sample groupings. Time point consideration revealed 28.4 % (566/1986) or 27.8 % (554/1986) circRNAs had an early (4 hr) or late (24 hr) expression pattern respectively.

**Figure 1:**
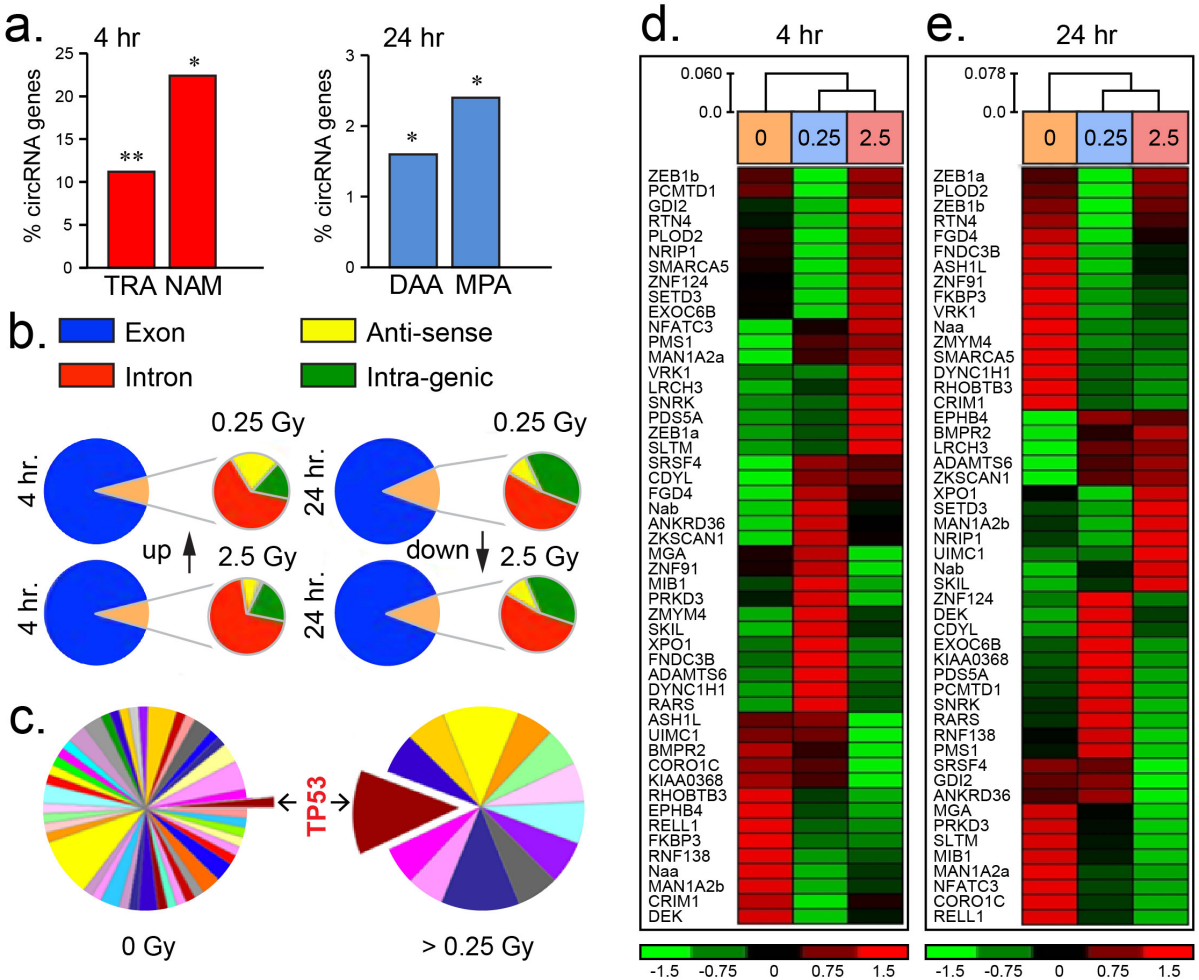
Differential circRNA expression post irradiation **(a)** Histograms of the percentage of circRNAs partaking in transcriptional regulator activity (TRA), nucleic acid metabolism (NAM), deacetylase activity (DAA) and metallopeptidase activity (MPA) compared to an expected threshold. **(b)** Pie-charts illustrating the predominance of circRNAs sourced from exonic regions of the human genome (hg19 assembly) post 4 and 24 hr low (0.25 Gy) or medium (2.5 Gy) dose irradiation. **(c)** Pie-charts showing increased number of circRNAs sourced from genes influencing TP53 (colour coded pathways are represented in Supplementary Table 1). **(d–e)** Heatmaps of circRNAs differentially expressed from fifty representative genes in endothelial HUVEC cells 4 or 24 hr post-irradiation. Expression level index shown below ranging from -1.5 to 1.5 fold change.

Radiation dose dependent circRNAs were found in 30.3 % (172/566) post 4 hr exposure (0.25 Gy – 2.5 Gy). These circRNAs were predominantly up-regulated (65.2 %; 112/172) with the remainder showing the opposite outcome (34.8 %; 60/172) 4 hr post irradiation. Bio-informatic assessment of significantly associated molecular functions over an expected threshold revealed that at this time-point circRNAs with enhanced expression were sourced from genes partaking in transcriptional regulator activity (11.2 %, p < 0.01) and nucleic acid metabolism (22.4 %, p = 0.04) (Figure 1a).

Radiation dose dependent circRNA expression was noted post 24 hr exposure with 35.3 % (196/554) of these transcripts being differentially altered. The majority of these circRNAs were upregulated (71.4 %; 140/196) at this time-point. Furthermore, post 24 hr circRNA producing genes significantly participated in deacetylase (1.6 %, p < 0.01) and metallopeptidase (2.4 %, p < 0.01) activity pathways over an expected threshold (Figure 1a). CircRNA were detected by the presence of exon-exon junctions within next generation sequencing traces using published criteria [22]. Radiation responsive circRNAs were sourced principally from transcripts emanating from exonic regions of the genome (Figure 1b). A subset of 50 representative circRNAs of known gene origin revealed highly variable transcript levels when samples were compared from irradiated or sham-irradiated HUVEC cells post 4 and 24 hr (Figure 1c–1e, Supplementary Figure 1). Within 24 hr following exposure to irradiation (ranging from 0.25 Gy to 2.5 Gy), circRNAs were predominantly produced from genes influencing the p53 pathway as well as others to a lesser degree (Figure 1c, Supplementary Figure 2). These initial findings demonstrate that circRNAs are dynamic transcripts which partake in the cellular response to low and medium dose irradiation exposure.

### Identification and verification of irradiation responsive *WWOX* circRNA

**Figure 2:**
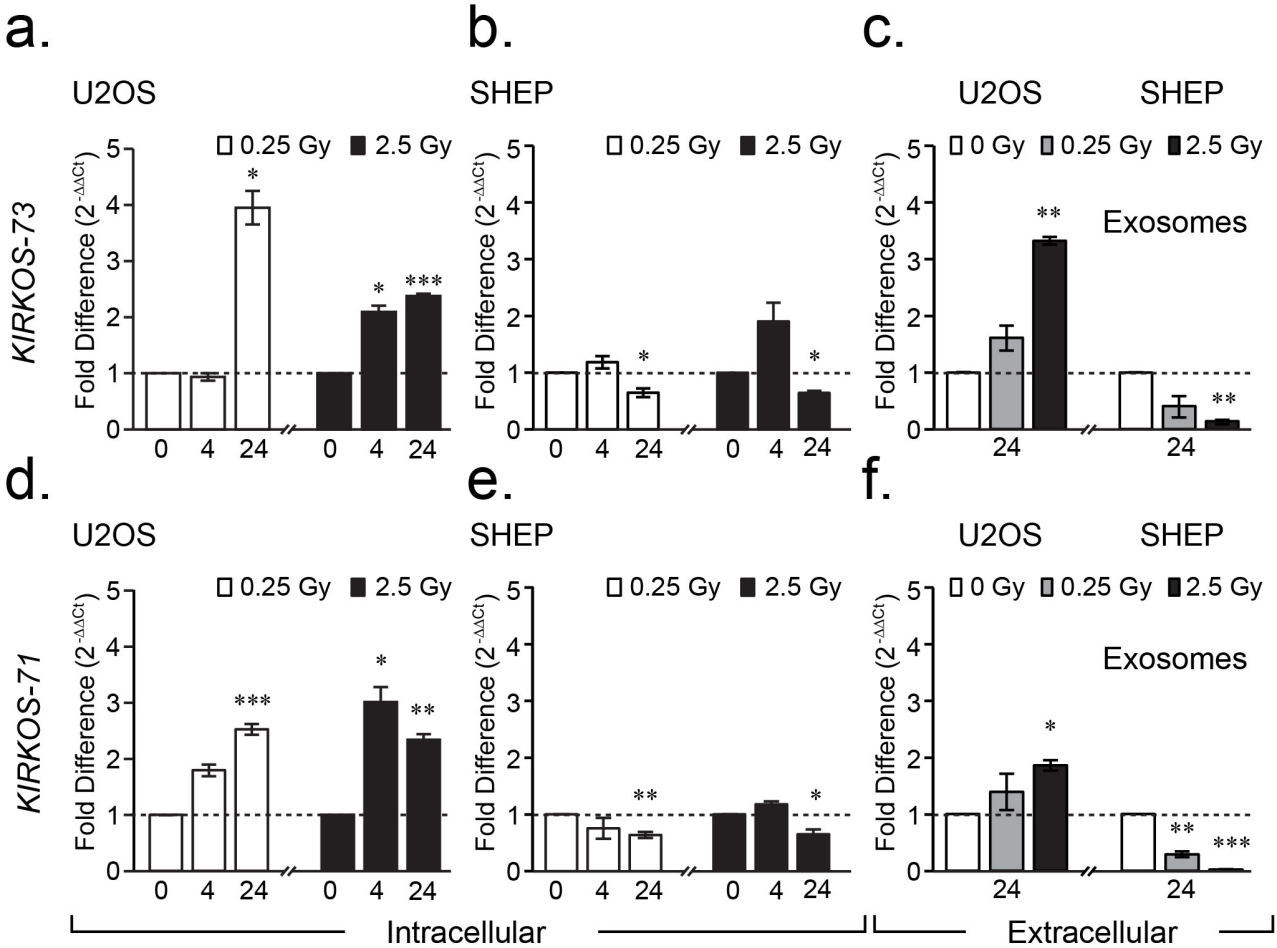
*WWOX* circRNAs *KIRKOS-71* and *KIRKOS-73* are irradiation responsive and enriched in exosomes **(a-f)** Histograms illustrating the differential fold change in *KIRKOS-73* (upper) and *KIRKOS-71* (lower) in U2OS **(a, d)**, SHEP **(b, e)** and exosomes from these cell lines **(c, f)** post irradiation (0.25 Gy (white) and 2.5 Gy (black)) in comparison to sham irradiated controls (represented by a dashed line). Asterisks represent significance, p < 0.05.

This investigation focused on two circRNAs *KIRKOS-73* and *KIRKOS-71* encoded by *WWOX* (tryptophan domain containing oxidoreductase), a known p53 regulator. Next generation sequencing data confirmed the presence of *KIRKOS-73* (451 bp; Circbase (http://www.circbase.org/) nomenclature hsa_circ_0040573) encoding *WWOX* exon 7 and 8 (Supplementary Figure 3) in a sham irradiated HUVEC endothelial cell line. Single assay design [1] and automated sequencing confirmed the presence of the back splice junction in *KIRKOS-73* (Supplementary Figure 3). *KIRKOS-73* was found to be significantly down-regulated in HUVEC 4 and 24 hr post low dose irradiation (0.69 ± 0.05 fold, p = 0.03; 0.74 ± 0.06 fold, p = 0.045 respectively) (Supplementary Figure 3a). Similarly, this circRNA was down-regulated in the neuroblastoma cell line SHEP 24 hr post low (0.58 ± 0.12 fold, p = 0.04) and medium (0.67 ± 0.2 fold, p = 0.01) dose exposure (Figure 2b). In contrast, *KIRKOS-73* showed a significantly elevated expression profile in the osteosarcoma cell line U2OS by 24 hr post 0.25 Gy in comparison to sham-irradiated control conditions (2.95 ± 0.29 fold increase, p = 0.01) (Figure 2a). This circRNA was also upregulated in this cell line post 4 hr (1.09 ± 0.11 fold increase, p = 0.01) and 24 hr (1.3 ± 0.03 fold increase, p = 0.0008) after 2.5 Gy exposure compared to controls (Figure 2a). According to CircNet (http://circnet.mbc.nctu.edu.tw/) *KIRKOS-73* is highly expressed in the human cervix, lung and skeletal muscles and up-regulated in embryonic stem cells (Supplementary Figure 3). In contrast, this circRNA is down-regulated in various other human tissues including adipose tissue, blood, liver, placenta and skin (Supplementary Figure 3).

While *KIRKOS-71* could not be detected in HUVEC, this *WWOX* circRNA (949 bp; Circbase hsa_circ_0040571) representing exons 2 – 8, was evident in SHEP and U2OS with single assay design [1] and automated sequencing confirmation of the presence of its back splice junction (Supplementary Figure 3). Down regulated transcript levels were detected for this circRNA particularly 24 hr post low (0.63 ± 0.05 fold, p = 0.002) and medium (0.64 ± 0.08 fold, p = 0.05) irradiation in SHEP (Figure 2e). In contrast, *KIRKOS-71* transcript levels were highly significantly increased in U2OS 24 hr post 0.25 Gy (1.52 ± 0.09 fold increase, p = 0.0001) in comparison to sham irradiated control cells (Figure 2d). Following medium dose exposure, this circRNA was similarly elevated post 4 hr and 24 hr (2.0 ± 0.26 fold increase, p = 0.017; 1.3 ± 0.1 fold increase, p = 0.006 respectively) compared to controls (Figure 2d). According to CircNet, *KIRKOS-71* is highly expressed in human embryonic stem cells and to a lesser extent in adipose tissue, liver and lungs (Supplementary Figure 3). In contrast, this circRNA is down regulated in human skeletal muscle embryonic stem cells with reduced expression in the liver, lung and monocytes (Supplementary Figure 3). These finding highlight the differential transcript levels of these *WWOX* circRNAs in various tissues and cell lines as well as their alternative response to irradiation.

### *WWOX* circRNAs *KIRKOS-71* and *KIRKOS-73* are present in exosomes and show differential transcript levels between cell lines post irradiation

Exosomes were extracted from SHEP and U2OS 24 hr following 2.5 Gy, 0.25 Gy or sham irradiation. *KIRKOS-73* and *KIRKOS-71* were found to be significantly upregulated in exosomes post 2.5 Gy in U2OS in comparison to their individual sham irradiated controls (p = 0.0008 and p = 0.011, respectively) (Figure 1c and 1f). In contrast, both of these circRNAs were significantly down regulated at this time point post 2.5 Gy in exosomes isolated from SHEP in comparison to sham irradiated controls (p = 0.002 and p = 0.000057, respectively) (Figure 1c and 1f). *KIRKOS-71* was also significantly down regulated 24 hr post 0.25 Gy in exosomes from this cell line in comparison to sham irradiated controls (p = 0.0048) (Figure 1f). These findings reveal that *KIRKOS-73* and *KIRKOS-71* are present extracellularly with contrasting clearance levels post irradiation.

### Cellular localisation of *KIRKOS-71*, *KIRKOS-73* circRNAs and the linear transcript

**Figure 3:**
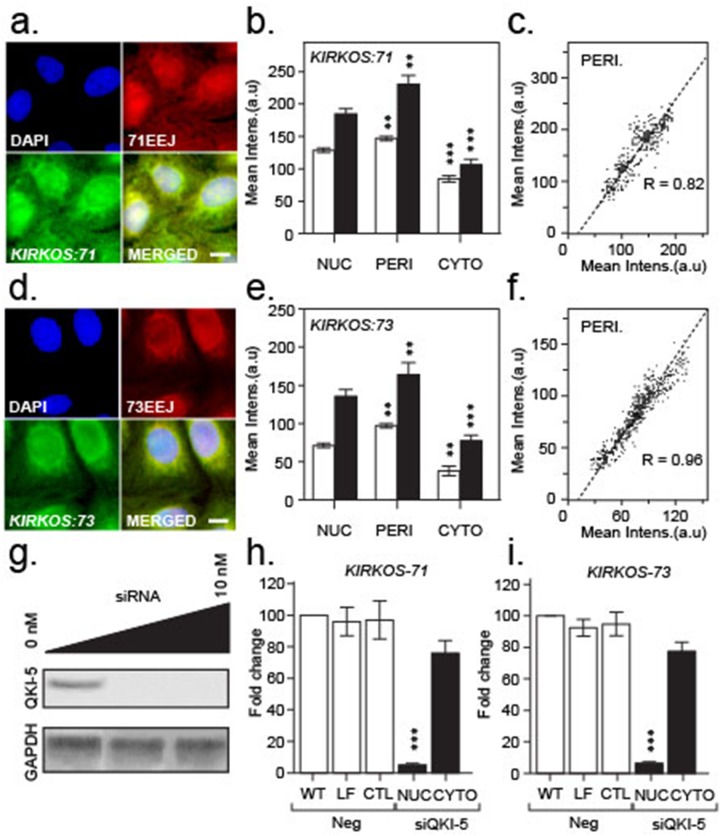
Intracellular localisation of *KIRKOS-71* and *KIRKOS-73* circRNAs with QK-5 depletion eliminating their nuclear presence **(a, d)** Representative epifluorescence micrographs of *KIRKOS-71* (a) and *KIRKOS-73* (d) identified with a specific Quasar 570 dual labelled probe against the exon-exon junction (EEJ; red) and Stellaris Fluorescein labelled FISH designed against the backbone (minus the exon-exon junction (green)). Merged images (yellow) and DAPI stained nuclei (blue) shown. Scale bar 20 μm. **(b, e)** Histograms of the mean intensity of Quasar 570 dual labelled probes (white) or Stellaris Fluorescein FISH (grey) bound to *KIRKOS-71* (b) or *KIRKOS-73* (e) in the nucleus (NUC), perinucleus (PERI) or cytoplasm (CYTO). Significance is represented by asterisks, p < 0.05. **(c, f)** Scatterplot of co-localization of mean intensity signals (arbitrary units (a.u)) from Quasar 570 dual labelled probes (x – axis) or Stellaris Fluorescein FISH (y - axis) bound to *KIRKOS-71* (c) or *KIRKOS-73* (f). **(g)** Representative Western blots of Quaking isoform 5 (QKI-5) plus or minus siRNA (0 to 10 nM) knockdown and GAPDH in U2OS. **(h, i)** Histograms of differential fold change in *KIRKOS-71* (h) and *KIRKOS-73* (i) in U2OS non-transfected (WT, nuclear samples shown) or transfected with lipofectamine (LF, nuclear samples shown), siRNA negative control (CTL, nuclear samples shown) or siRNA against QKI-5 (10 nM; nuclear and cytoplasmic samples shown). Significance is represented by asterisks, p < 0.05, n = 3.

The sequence within the circRNA exon-exon junction enabled dual end-labelled oligonucleotides to be designed that specifically annealed to *KIRKOS-71* or *KIRKOS-73*. Furthermore, Stellaris FISH Fluorescein labelled probes were produced against the circRNA backbone (minus the exon-exon junction). The combination of these two detection systems assisted in determining the intercellular distribution of these circRNAs. The mean intensity of the FISH probes (green emission) was on average 28.9 ± 4.5 % and 44.2 ± 3.1 % greater than the Quasar labelled oligonucleotides (red emission) binding to *KIRKOS-71* and *KIRKOS-73* respectively, as expected due to the increased presence of relevant probe binding sites. The greatest signal intensity for both of these circRNAs in U2OS was in the perinuclear region (defined in materials and methods), 24 hr post low dose irradiation exposure compared to the nucleus (Quasar 570 emission: *KIRKOS-71* p = 0.0058 ; *KIRKOS-73* p = 0.0012) or cytosol (Quasar 570 emission: *KIRKOS-71* p = 0.0001 ; *KIRKOS-73* p = 0.003) (Figure 3a–3b, 3d–3f). High levels of co-localization were determined for the detection systems used for these circRNAs (Figure 3c and 3f, Supplementary Table 1). These findings demonstrate that these distinct circRNAs display similar intercellular expression pattern after irradiation exposure.

The intracellular distribution of a linear *WWOX* mRNA transcript (*linWWOX*; representing exons 1 – 9, Supplementary Figure 4) was determined for comparison purposes against the *KIRKOS-71* and *KIRKOS-73* U2OS cellular profile. As exon 1 and 9 are absent from these circRNAs, probes were designed to specifically detect *linWWOX* (Supplementary Table 2). In contrast to *KIRKOS-71* and *KIRKOS-73*, the highest mean intensity for probes specific for *linWWOX* was in the nucleus/nucleolus. The *linWWOX* transcript was also found in the perinuclear region and in the cytosol but to a lesser extent compared to *KIRKOS-71* and *KIRKOS-73* (Supplementary Figure 4). These results show that the *linWWOX* is expressed throughout U2OS with a dissimilar distribution pattern to *KIRKOS-71* or *KIRKOS-73*.

### Depletion of QKI isoform 5 eliminates nuclear *KIRKOS-71* and *KIRKOS-73* but spared their presence in the cytosol

Translation of QKI-5 was heavily compromised following its targeting via siRNA transfection in U2OS (Figure 3G). Intercellular compartment fractionation post transfection revealed that depletion of QKI-isoform 5 eliminated *KIRKOS-71* (p = 0.0003) and *KIRKOS-73* (p = 0.0004) in the nucleus but not significantly in the cytosol (Figure 3H and 3I) compared to negative controls. This result suggests that QKI-5 is responsible for *de novo* synthesis/splicing of these circRNA in the nucleus with rapid export to the cytosolic compartment. No significant difference between WT (and controls) compared to KD demonstrates that cytosolic *KIRKOS-71* and *KIRKOS-73* are most likely QKI-5 independent.

### QKI isoforms differ in their intracellular distribution

**Figure 4:**
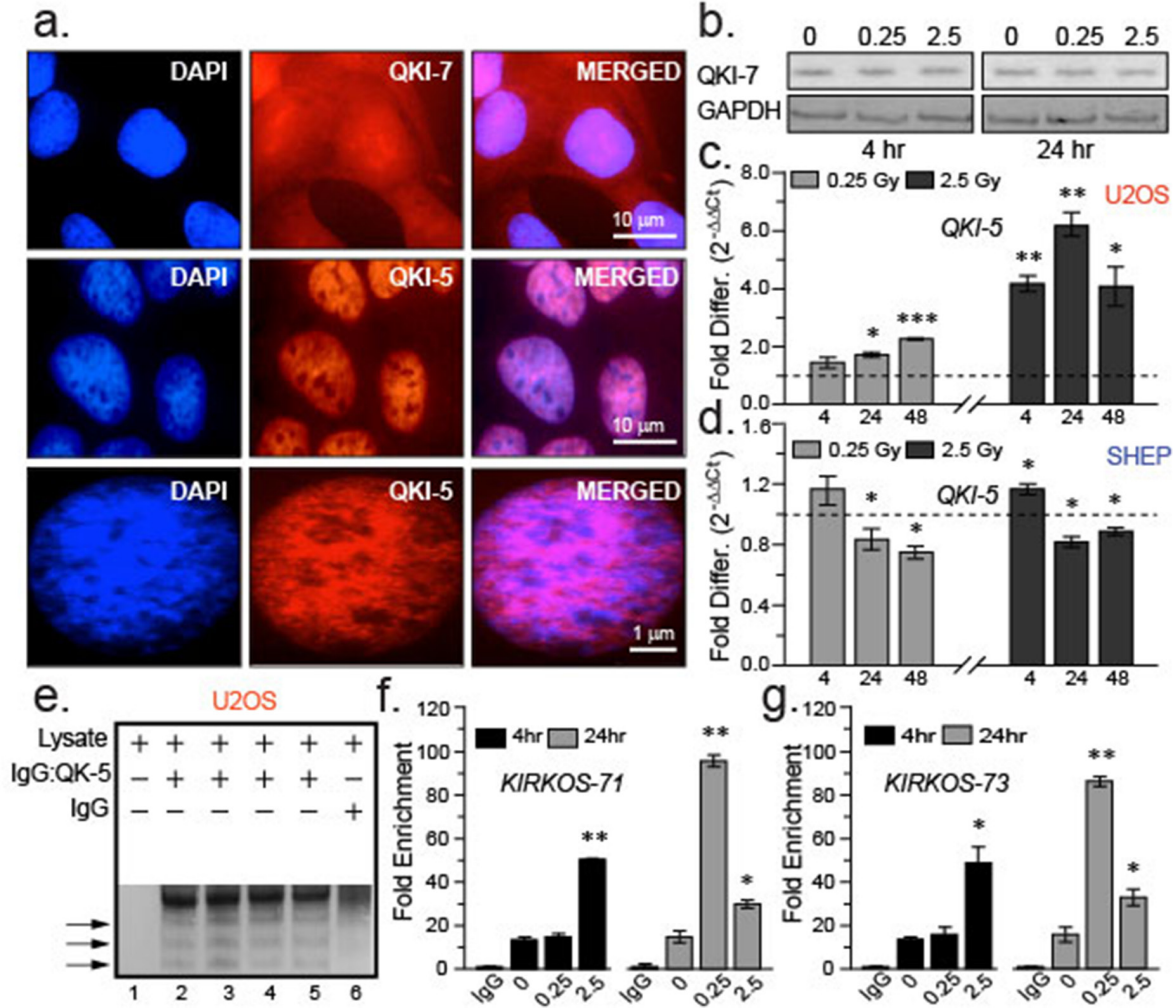
QKI isoforms differ in their intracellular distribution and response to irradiation exposure **(a)** Representative epifluorescence micrographs of immuno-stained QKI-7 (red, top) or QKI-5 (red, middle and lower) in U2OS. Merged images (right) and DAPI stained nuclei (left) shown. Scale bars are 10 μm (upper and middle images) and 1 μm (lower) respectively. **(b)** Representative Western blots of QKI-7 and endogenous control GAPDH in U2OS cell lysates 4 and 24 hr post sham irradiation or irradiation (0.25 Gy and 2.5 Gy). **(c, d)** Histograms of fold changes in QKI-5 transcript levels post irradiation compared to sham irradiation (dashed line) in U2OS (c) or SHEP (d). *KIRKOS-71* and *KIRKOS-73* associate with QKI-5 in a radiation responsive manner. **(e)** Representative NuPage Bis Tris gel of U2OS pull down fractions in the presence or absence of QKI-5 specific antibody or IgG control. **(f, g)** Histograms of fold enrichment of *KIRKOS-71* (f) and *KIRKOS-73* (g) associated with QKI-5 4 or 24 hr post irradiation. Significance is represented by asterisks, p < 0.05, n = 3.

The presence of QKI isoform 5 (QKI-5) was demonstrated through immuno-histochemical analysis of U20S. QKI-5 was found predominantly in the nucleus of U2OS yet strikingly absent from nucleoli (the location of ribosome synthesis and assembly) in this cell line in comparison to another QKI isoform 7 (QKI-7) which appeared distributed throughout the cell (Figure 4a). While the expression of QKI-7 appeared unaltered post low or medium dose exposure (Figure 4b), a significant increase of *QKI-5* expression was detected 24 hr (p = 0.0121) following 0.25 Gy. Similarly, *QKI-5* was augmented 24 hr after medium exposure (4 hr, p = 0.0035; 24 hr, p = 0.0047) compared to sham-irradiated controls (Figure 4c). In contrast, transcription of *QKI-5* decreased 24 hr and 48 hr post low and medium dose irradiation exposure in the SHEP cell line (Figure 4d). These finding reveal parallel transcriptional trends between *QKI-5, KIRKOS-71* and *KIRKOS-73* in these cell lines indicative of their potential association.

### *KIRKOS -71* and *KIRKOS -73* associate with QKI-5 in a radiation responsive manner

Due to reduced levels of *QKI-5* expression in SHEP post irradiation (Figure 4d), RNA immunoprecipitation of endogenously formed circRNA: QKI-5 complexes was determined in irradiated and sham-irradiated U2OS. The antibody pull down specificity for QKI-5 was established in this cell line with comparison to an IgG control fraction (Figure 4e). QPCR amplification confirmed the presence of the *KIRKOS-71* and *KIRKOS-73* associating with QKI-5 in U2OS (Figure 4f-4g). At 24 hr post low or medium dose irradiation exposure an escalation in *KIRKOS-71*: QKI-5 linkage was noted particularly after 0.25 Gy (81.8 ± 2 fold increase, p = 0.001; Figure 4f). Increased association of *KIRKOS-73* with QKI-5 was noted 4 hr post medium dose irradiation exposure (34.6 ± 6 fold increase, p = 0.03; Figure 4g). At the 24 hr time point post low dose irradiation this association increased very significantly (69.6 ± 2 fold increase, p = 0.002) and to a lesser extent after 2.5 Gy exposure (15.58 ± 3 fold increase, p = 0.05; Figure 4g). These findings reveal that in response to irradiation exposure a cooperative interaction between QKI-5 and *WWOX* tumor suppressor circRNAs is instigated.

## DISCUSSION

**Figure 5:**
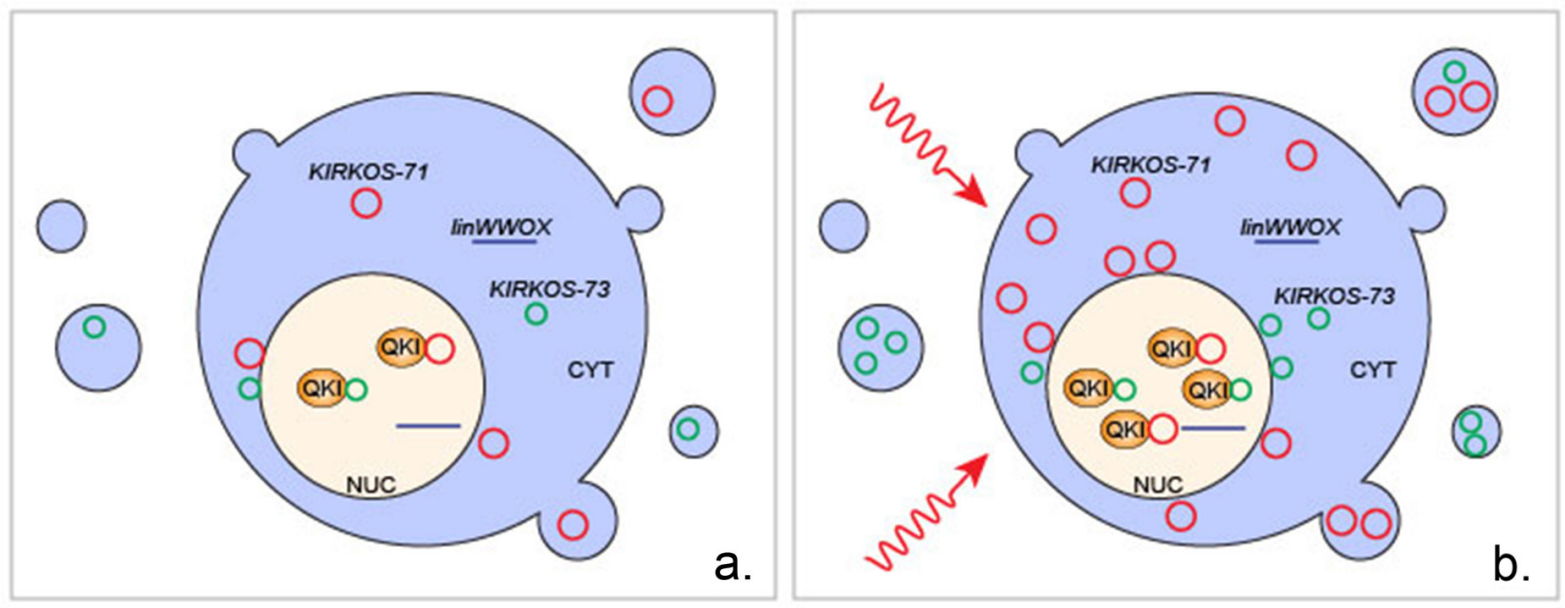
Overview of the proposed intercellular and extracellular response of circRNAs *KIRKOS-71* and *KIRKOS-73* as well as the QKI isoform 5 protein to irradiation exposure **(a)** *KIRKOS-71* (red circle) and *KIRKOS-73* (green circle) are present in the nucleus (NUC), cytoplasm (CYT) and in exosomes in a non-irradiated cell. **(b)** Within 24 hr post low and medium dose irradiation exposure increased expression of *KIRKOS-71*, *KIRKOS-73* and QKI isoform 5 (QKI) occurs along with their increased association in the osteosarcoma cell line U2OS. Linear *WWOX* transcript (*linWWOX*).

This study provides the first comprehensive report that unveils circRNAs as cellular responders to ionising radiation. Post exposure, we found that circRNAs prominently emanate from genes influencing p53, the so called ‘the guardian of the genome’ due to its role in conserving gene stability [23]. While the current literature is sparse in descriptive studies of individual circRNAs, we hereby provide the introduction to *KIRKOS-71* and *KIRKOS-73*, two heterogeneous ‘circles’ spliced from *WWOX*, the p53 stabiliser. Varied circRNA expression between cell types [3] is supported here with their varied clearance levels within exosomes in response to irradiation (Figure 5). The RNA binding protein QKI, a chief modulator of circRNA biogenesis [24], revealed isoform specific expression profiling and association with *WWOX* circRNAs contingent to irradiation exposure.

It has been reported that circRNAs source from almost a quarter of actively transcribed human genes [25]. CircRNAs may regulate cellular stress in attempts to maintain homeostasis or boost adaptation to environmental challenges [26]. While the focus has been placed recently on lncRNA and miRNA [20, 21], a knowledge gap has remained to date as to whether circRNA react to ionizing radiation (IR). An initial exploratory phase using human endothelial cells revealed 65% of the circRNA on a microarray were differentially expressed post low and medium dose exposure. Similar to other independent reports this analysis revealed that circRNAs expression patterns appeared to be globally variable across time and dose within a single cell type [27]. Nevertheless, diverse early and late effects became evident with enrichment of circRNAs spliced from genes partaking in transcription regulator activity and nucleic acid metabolism. Deacetylase activity was linked to genes producing differentially regulated circRNAs by 24 hr after such irradiation conditions in comparison to reports that examined other stimuli eg. epidermal growth factor (EGF) [28]. Metallopeptidase activity was also identified for this sub-group similar to another large RNA sequencing study in which circRNA-CER (hsa-circ0023404) was identified [1]. Detailed characterization of this circRNA revealed its potential as a miRNA sponge, important for regulation of MMP13 (matrix metallopeptidase 13) [29]. Differentially expressed circRNA appeared to arise predominantly from back-spliced exonic regions of the genome post irradiation. Our primary investigation for irradiation responsive circRNAs indicated a strong bias for genes influencing the pivotal IR checkpoint protein of mammalian cells - TP53 [30].

The first WWOX partner to be identified in mouse was the *p53* homolog, p73 [19]. Ectopic expression of wild type WWOX preferentially induced apoptosis in human glioblastoma cells harbouring mutant p53 [31]. Activated WWOX physically interacts with serine 46-phosphorylated p53 instigating stability and apoptotic functionality [32]. Herein, we demonstrated the diverse expression profile of *WWOX* circRNAs *KIRKOS-71* and *KIRKOS-73* in response to irradiation. While *KIRKOS-71* was non-detectable, *KIRKOS-73* exhibited diminishing transcript levels post irradiation in HUVEC in comparison to sham-irradiated controls. Further analysis noted a similar intracellular and extracellular reduction for both circRNAs in the neuroblastoma cell line SHEP 24 hr post 0.25 Gy and 2.5 Gy. In contrast, *KIRKOS-71* and *KIRKOS-73* became significantly upregulated in the osteosarcoma cell line U2OS 24 hr post low dose irradiation and by 4 hr after medium exposure. Their increased transcript levels were furthermore reflected in their augmented presence in extracellular exosomes 24 hr post-irradiation. Previously, it has been shown that circRNAs co-precipitate with extracellular vesicles in supportive evidence for their expulsion from cells and as a potential means of functional communication [14, 33]. *KIRKOS-71* and *KIRKOS-73* were enriched in the perinuclear region of U2OS in comparison to their linear transcript. CircRNA *Zeb1* has also been identified in the perinucleus in embryonic porcine cells with an exclusive nuclear location upon maturation [34].

The RNA binding protein Quaking (QKI isoform 5) is a major regulator of circRNA biogenesis during epithelial to mesenchymal transition [16]. Our results support previous findings of the restricted nuclear predominance of QKI-5 in contrast with the widespread intracellular distribution of QKI-7 [35]. QKI-7 known as the apoptotic inducer becomes suppressed by heterodimer formation with other QKI isoforms leading to nuclear translocation [36]. Limited knowledge exists to date in relation to the effects of ionising radiation on QKI. In *C. elegans*, a genetic screen to identify mutants displaying apoptosis following ionizing radiation led to the identification of GLD-1 (GermLine Development defective), a homolog of human QKI [37]. Our findings reveal that QKI isoform 5 and 7 have distinct reactions to irradiation. QKI-7 expression was found to be unaltered yet QK-5 significantly increased in U2OS 24 hr after low and medium dose exposure. In contrast, QKI-5 significantly diminished in SHEP by this time point following such conditions. The influence of nuclear QKI-5 on *WWOX* circRNA expression was apparent as both *KIRKOS-73* and *KIRKOS-71* were very significantly decreased in the nucleus upon its knockdown. The significantly increased association of *KIRKOS-71* and *KIRKOS-73* with QKI-5 post low and medium dose irradiation found in this study reflects their ability to act as potential mutual modulators responding to cellular stress.

The implications for the conjoint relationship between QKI and circRNAs will no doubt influence resistance in the presence of malignancy or infection. While the functions and related mechanisms of circRNAs and their interacting partners have yet to be deciphered, it can be ventured that these are most likely diverse and of impending importance within the spectrum of therapeutic intervention.

## MATERIALS AND METHODS

### *In vitro* propagation of cell lines

Human Umbilical Vein Endothelial (HUVEC) were propagated in Medium 200 supplemented with 1 × LSGS (Low Serum Growth Supplemented) and purchased from Cascade Biologics, Inc. (Portland, OR). Osteosarcoma (U2OS) were cultured in Dulbecco’s Modified Eagle Medium (DMEM) with high glucose (cat. # 11960 -044, Life Technologies). Neuroblastoma (SHEP) were propagated in Roswell Park Memorial Institute (RPMI) 1640 Medium and GlutaMAX Supplement (cat. # 61870 - 036, Life Technologies). Both U2OS and SHEP media were supplemented with 10 % Fetal Bovine Serum (FBS), penicillin and streptomycin (1%, Sigma). All cells were cultured at 37°C in a humidified incubator containing 95 % oxygen/5 % carbon dioxide atmosphere. HUVEC were utilised at passage no.6 before initiation of senescence/differentiation. The identity of all cell lines was verified by microsatellite analysis (Eurofin Medigenomix, Forensik GmbH, Germany). All cultures were routinely checked for mycoplasma contamination using a MycoAlert Mycoplasma detection kit (Lonza, cat. # LT07-218). In general, cells were grown to 80% confluency prior to removal from the dish using trypsin (0.25%)/EDTA (0.02%) and sub-culturing or harvesting.

### Irradiation

All irradiation were performed using a closed HWM-D 2000 Cesium^137^ source (Wälischmiller Engineering GmbH, Markdorf, DE; 10cm height, 33cm diameter) at a dose rate of 0.0082Gy/sec. Cells were exposed to low (0.25 Gy) or medium (2.5 Gy) doses. Sham irradiation of controls involved only transport to the irradiation facility. Annual calibration was performed by the Helmholtz Zentrum Munich, DE with reference to standards established by the National Physical Laboratory (U.K).

### Exosomal isolation

For the isolation of exosomes from SHEP and U2OS, 1 × 10^7^ cells (per dose) were seeded in the appropriate growth media (indicated above) which was replaced 24 h later by exosome-free media (growth media post removal of bovine exosomes from FBS using overnight centrifugation at 100,000 g). Cells were sham-irradiated or irradiated at 0.25 or 2,5 Gy. Exosome isolation was performed 24h later according to a previous protocol [38]. In brief, cell culture supernatants were centrifuged at 10,000 g for 30 min, filtered through a 0.22 μm pore size filter for cell debris and larger vesicle removal. Exosomes were sedimented from the filtrate by ultracentrifugation at 100,000 g for 75 min at 4°C. The exosomal pellet was resuspended in 2 ml PBS. After repetition of the ultracentrifugation step the supernatant was discarded and the exosomes re-suspended in 200 μl PBS and stored at -20°C.

### RNA isolation

Total RNA was isolated from exosomes using a *mir*Vana™ miRNA isolation kit (Ambion RNA Life Technologies, cat. # AM1560). Note, this kit enables total RNA to be extracted and while the option for further purification of RNA enriched for small RNAs is possible, this was not carried out for this study. In brief, exosomes resuspended in PBS (see above) were disrupted in lysis solution, RNA extracted with phenol/chloroform with ethanol precipitation. Solid phase filter cartridge RNA purification was carried out using appropriate washing solutions with final elution in ultra-pure water (heated to 95°C) with concentration and purity assessment using O.D. 260/280 ratio determination (NanoDrop 1000, Thermo Scientific).

Total RNA was isolated from cell lines and purified using TriFast peqGOLD (Peqlab, cat # 30 – 2010) and a Maxwell® 16 LEV Blood DNA kit (Promega cat # AS1290) with solution substitution (ie. isopropanol replacement by 100 % ethanol in cartridge number 1) and Maxwell® 16 machine (Promega). Final elution was in ultra-pure water with concentration and purity assessment as outlined above. In addition, the RNA integrity number (RIN) was measured using a bioanalyzer machine (2100 Bioanalyzer). Values higher than 7.00 demonstrated a high quality of RNA.Total RNA was stored at -80°C.

### Microarray circRNA expression profiling

Total RNA from irradiated (0.25 Gy or 2.5 Gy) or sham irradiated HUVEC was commercially outsourced for circRNA expression profiling on an Arraystar Human circRNA Array (6x7K. Arraystar Inc). Quantile normalization of raw data and subsequent data processing were performed using the R software package. Comparative analysis of circRNA expression profiles between sample sets having a fold change > 2 and a p-value < 0.05 were considered significantly differentially expressed.

### Bioinformatic analysis of circRNA expression

Bioinformatics software was utilised for data analysis (BaseSpace, Illumina Inc.) and interpretation of the Human circRNA array (6x7 K,Arraystar). Excel data sheets provided a source for obtaining the circRNA probe information and was used for data mining via circBase (http://circBase.org).

### cDNA synthesis

Total RNA (1μg) from non-irradiated and irradiated cells was converted into first strand cDNA using standard protocol procedures (with the inclusion of random hexamers or oligo dT primers) and reagents from Life Technologies, Germany.

### Real time PCR quantification

*KIRKOS-71* and *KIRKOS-73* expression was measured using divergent primers and reporter quencher-labelled probes manufactured by Eurofins Genomics GmbH, Ebersberg (Sequences listed in Supplementary Table 1). *TBP* primers and probes (O’Leary et al 2015) or pre-designed *QKI* (Hs00287641_ml) single Taqman gene expression assays (ThermoFisher cat # 4331182) were utilized for gene expression determination. The reaction conditions for single gene assays were as such: cDNA (50 - 100 ng), 1xTaqman universal PCR master mix (no AmpErase UNG; Life Technologies, cat. # 4324018), forward and reverse primers (10pmol), specific fluorescent probe (5pmol), nuclease-free water up to 25μl. For pre-designed assays similar conditions were used except for assay mix (1 X) utilized instead of individual primers and probes. Amplification conditions were prepared as per manufacturer instructions (Life Technologies, cat # 4384556) and performed as follows: 95°C 10 min (1 cycle), 95°C 15 sec and 60°C 1 min (40 cycles), 4°C hold.

### RNA interference targeting *QUAKING* isoform 5

*QKI-5* knockdown was undertaken with Mission esiRNA (cat. # EHU090851, Sigma) interference technology. U2OS and SHEP were grown to 50% confluence and transfected with differential volumes of these esiRNAs (200 ng/μL) (0.5 μL; 2.5 μL, 5 μL) using lipofectamine as per manufacturer instructions. After 72 hr, cells were irradiated at 0.25Gy, 2.5Gy or sham-irradiated (0 Gy). Control conditions included sham irradiation plus transfection with lipofectamine. RNA extraction was performed 4 hr and 24 hr post irradiation (or sham irradiation).

### Nuclear isolation

Nuclei were isolated from U2OS using a nuclear extraction kit (Millipore cat # 2900). In brief, cells were grown to 70 – 90% confluency and removed by trypsinization following standard protocols. Cell pellets (2 × 10^6^ cells) were resuspended in cytoplasmic lysis buffer (500μl) containing 0.5 mM DTT and protease inhibitor cocktail (1 in 1000 dilution) with incubation on ice for 15 min. Following centrifugation at 250 g for 5 min at 4°C, the supernatant was discarded and cell pellet resuspended again in cytoplasmic lysis buffer (200 μl). Cell lysis was performed by drawing the cell suspension through a 27-gauge needle. Following centrifugation at 8,000 g for 20 min at 4°C, the nuclear pellet was resuspended in nuclear extraction buffer (70 μl) containing 0.5 mM DTT and protease inhibitor cocktail (1 in 1000 dilution). Nuclei were disrupted via passage through a 27-gauge needle and incubation for 60 min at 4°C. Following centrifugation at 16,000 g for 5 min at 4°C, the supernatant representing the nuclear extract was obtained.

### Protein extraction

Protein extraction from SHEP and U2OS cells was performed +/- QKI knockdown using a T-PER reagent (cat. # 78510, Thermo Scientific) with addition of protease inhibitor cocktail tablets (cat. # 04693116001, Roche). 100 μL of the reagent was added to the 1 × 10^6^ cells followed by homogenization and sonication (20 sec) for membrane disruption. Cells were centrifuged for 5 min at 10,000 × g to remove cell debris. Protein concentration was determined using a bicinchoninic acid (BCA) assay (cat. # 23227, Thermo Scientific).

### Electrophoresis and Western blotting

Cell lysates (10 μL) were mixed with 4 × NuPage LDS Sample Buffer (Life Technologies) (2.5 μL) and heated for 5 min at 70°C before loading onto 12 % Bis Tris NuPage gels (cat. # NP0342BOX, Novex Life Technologies) with electrophoresis in 1 × MOPS - SDS running buffer at 180 V in 4°C. Separated proteins were transferred onto Nytran membranes under standard conditions followed by blocking in 5 % bovine serum albumin in TBS-T. Detection of QKI-isoform 5 or QKI-isoform 7 was determined with over-night incubation at 4°C (1:500 in blocking reagent) using rabbit anti-QK-5 monoclonal antibody (mAb) (cat. # ab126742, abcam) or rabbit anti-QK-7 mAb. (cat. # XQKI-7, Millipore). GAPDH was also detected as a comparative control using a mouse anti-GAPDH mAb (cat. # C2514, Santa Cruz Biotechnology). Following extensive washing with TBS-T, nytran membranes were exposed to alkaline phosphatase-conjugated goat-anti rabbit (1:1000) (cat. # A-3687, Sigma Aldrich) or goat-anti-mouse (1:10000) (cat. # A-3562, Sigma) secondary antibody for 1 hr at RT. Specific proteins (QKI-5 and GAPDH) were visualized using a mix of 5-bromo-4-chloro-3’-indolyl-phospate p-toluidine and nitro-blue tetrazolium chloride solution (cat. # 1001973039, Sigma Aldrich). Western blots were photographed using a FluorChem HD2 gel visualization system (Alpha Innotec, Germany) with specific protein band intensities quantified using ImageJ (NIH, Bethesda, MD, USA).

### Fluorescence *in situ* hybridization and confocal microscopic analysis

Procedures were followed according to the Stellaris fluorescence *in situ* hybridization (FISH) (Biosearch Technologies) website (www.biocat.com). Using the online probe designer tool (www.biosearchtech.com/stellarisdesigner/), 25 specific probes were selected from an input sequence (hsa_circ_0040571 or hsa_circ_0040573 from Circbase (http://www.circbase.org)) without the region covering the exon exon junction (40 base pairs). Search parameters were selected that included a masking level (3), maximum probe coverage = 25 and minimum 1 nucleotide spacing level. Probe fluorophore 5’carboxyfluorescein FAM (Excitation (Ex): 495 nm; Emission (Em): 520 nm) were used for circRNA backbone detection. Custom oligonucleotide probes were commercially synthesised against the exon exon junction sequences (40 base pairs) of *KIRKOS-71* and *KIRKOS-73* dual end labelled with Quasar®570 (Ex: 552 nm; Em: 570 nm). Images were acquired using a GFP and TexasRed filter wheel on an inverted Axiovert 200 (Zeiss) fluorescence microscope with apotome slide module activation. Colocalisation and mean intensity analysis were performed with FIGI software (NIH). The perinuclear region was defined as a 3μm zone surrounding the nucleus. Co-localization of Fluorescein and Quasar®570 labelled probes was defined by the presence of these two labels in the same pixel in the digitally acquired images, using a co-localization algorithm (Zen 2008, Carl Zeiss). Separation of emission spectra was ensured with appropriate cut-off filters (green 492 - 590 nm; red 585 - 734 nm).

### Quaking immunofluorescent staining

U2OS were grown to ∼ 70 % confluence on glass-bottomed 35 mm dishes (uncoated, Ibidi GmbH) and fixed with 4 % paraformaldehyde (PFA) for 30 min followed by a 1 × PBS wash. Cells were permeabilized with PBS-T (0.5 % triton X-100) for 1 hr at RT and blocked for 1 hr in 5 % BSA in 1 × PBS-T supplemented with 2 % goat-serum. Cells were then exposed over-night at 4°C to appropriate QKI primary antibodies (1:500 in blocking reagent: anti-QK-5 monoclonal antibody (mAb) (1:500 in blocking reagent; cat. # ab126742, abcam) or rabbit anti-QK-7 polyclonal antibody (pAb) (1:500; cat. # AB9908, MerckMillipore). Following extensive washing steps (three times for 15 min in 1 × PBS), cells were exposed to secondary Ab labeled with Alexa Fluor 568 goat-anti rabbit (1:1000 in blocking reagent; cat. # A11011, Life Technologies) with incubation for 1 hr at RT. Control dishes were incubated with secondary Ab only. Following additional washing steps performed as described above, cells were mounted in hardset Vectrashield with DAPI (cat. # H-1500, Vectorlabs) and covered with cover slips. Confocal fluorescence images were obtained using a Keyence confocal microscope (# BZ-9000, Biorevo) with associated BZ II viewer software. QK-5 or QK-7 was visualised through excitation of the Alexa fluor 568 nm laser and image acquisition using the appropriate filter wheel.

### *KIRKOS-71* and *KIRKOS-73* binding to QKI

RNA-binding protein immunoprecipitation (RIP) was conducted using a Magna RIP kit (cat. # 17-700 Merck) as per manufacturer instructions. RNA was isolated from the retained supernatant as outlined above. A negative control was prepared alongside which represented the sample but with the exception of exchanging the primary Ab for a rabbit-anti-IgG Ab. The presence or absence of circRNA was determined by QPCR as outlined above with analysis undertaken using the online data analysis calculator (http://www.sigmaaldrich.com/life-science/epigenetics/imprint-rna.html).

### Statistical analysis

Statistical analyses were applied using two-sided student T-test with significance determined as a p-value < 0.05.

## SUPPLEMENTARY MATERIALS FIGURES AND TABLES




