# Supplementary material for: The circRNA interactome–innovative hallmarks of the intra- and extracellular radiation response
Source: Oncotarget. 2017 Jul 13;8(45):78397–409. doi: 10.18632/oncotarget.19228 (PMC5667970; doi:10.18632/oncotarget.19228)
Supplement: Supplementary file 1 [file oncotarget-08-78397-s001.pdf]

The circRNA interactome–innovative hallmarks of the intra- and extracellular radiation response

SUPPLEMENTARY MATERIALS

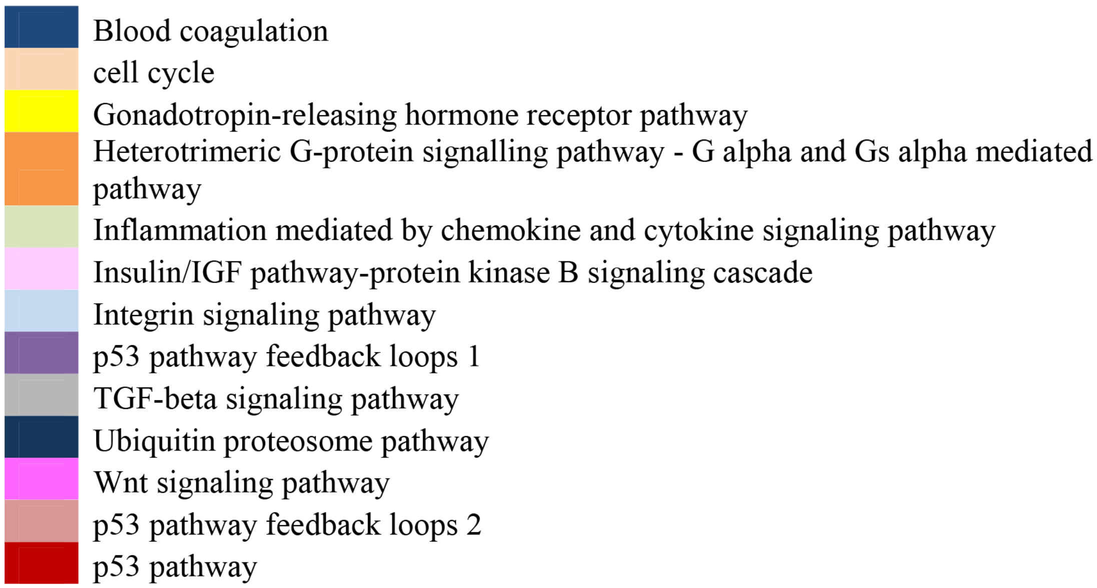

Supplementary Figure 1 related to Figure 1: Molecular pathways associated with circRNAs expressed in the endothelial cell line HUVEC 24 hr post sham-irradiation.

- 5HT1 type receptor mediated signaling pathway
- 5HT2 type receptor mediated signaling pathway
- 5HT3 type receptor mediated signaling pathway
- 5HT4 type receptor mediated signaling pathway
- ATP synthesis
- Adrenaline and noradrenaline biosynthesis
- Alpha adrenergic receptor signaling pathway
- Angiogenesis
- Axon guidance mediated by netrin
- Beta cell activation
- Beta1 adrenergic receptor signaling pathway
- Beta2 adrenergic receptor signaling pathway
- Beta3 adrenergic receptor signaling pathway
- CCKR signaling map
- Corticotropin releasing factor receptor signaling pathway
- Dopamine receptor mediated signaling pathway
- EGF receptor signaling pathway
- EGF signaling pathway
- Fructose galactose metabolism
- Gonadotropin-releasing hormone receptor pathway
- Heterotrimeric G-protein signaling pathway - G alpha and Gs alpha mediated pathway
- Huntington disease
- Inflammation mediated by chemokine and cytokine signaling pathway
- Insulin/IGF pathway-protein kinase beta signaling cascade
- Integrin signaling pathway
- Ionotropic glutamate receptor pathway
- Metabotropic glutamate receptor group II pathway
- Metabotropic glutamate receptor group III pathway
- Muscarinic acetylcholine receptor 1 and 3 signaling pathway
- Muscarinic acetylcholine receptor 2 and 4 signaling pathway
- Nicotinic acetylcholine receptor signaling pathway
- Notch signaling pathway
- Opiod prodymorphin pathway
- Opiod proenkephalin pathway
- Opiod proopiomelanocortin pathway
- Oxytocin receptor mediated signaling pathway
- p53 pathway feedback loops 1
- Parkinson disease
- Synaptic vesicle trafficking
- T cell activation
- TGF-beta signaling pathway
- Thyrotropin-releasing hormone receptor signaling pathway
- Ubiquitin proteosome pathway
- VEGF signaling pathway
- Wnt signaling pathway
- p53 pathway feedback loops 2
- p53 pathway

Supplementary Figure 2 related to Figure 1: Molecular pathways associated with circRNAs expressed in the endothelial cell line HUVEC 24 hr post irradiation (> 0.25 Gy - 2.5 Gy).

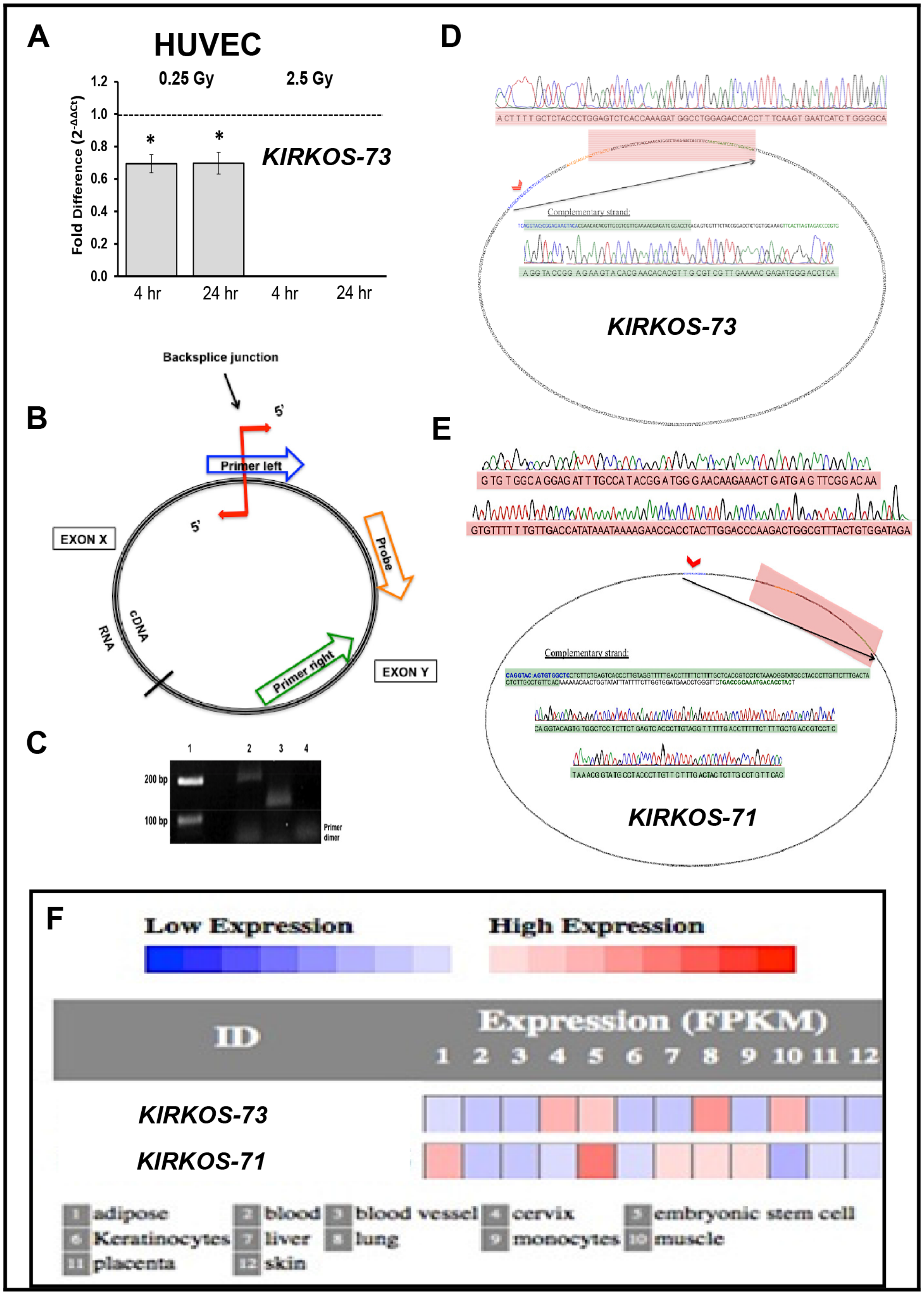

Supplementary Figure 3 related to Figure 2: (A) Quantitative PCR (QPCR) expression of *KIRKOS-73* in the endothelial cell line HUVEC 4 and 24 hr post 0.25 Gy or 2.5 Gy compared to sham irradiated controls (dashed line). All data were normalised to 1 against the TATA-binding protein (TBP) as an internal endogenous control. **(B)** Schematic of the Taqman QPCR assay design strategy. **(C)** Agarose gel electrophoresis (cropped image) of PCR circRNA product amplification. Lane 1: 1 Kb ladder (Biorad); lanes 2 and 3: PCR products of amplified regions from *KIRKOS-71* and *KIRKOS-73* respectively; lanes 4: no-template control. The presence of primer-dimers is indicated. **(D - E)** Automated sequencing traces of *KIRKOS-73* and *KIRKOS-71*. **(F)** Expression heatmap of *KIRKOS-73* and *KIRKOS-71* in various human tissue (CircNet (http://circnet.mbc.nctu.edu.tw/)).

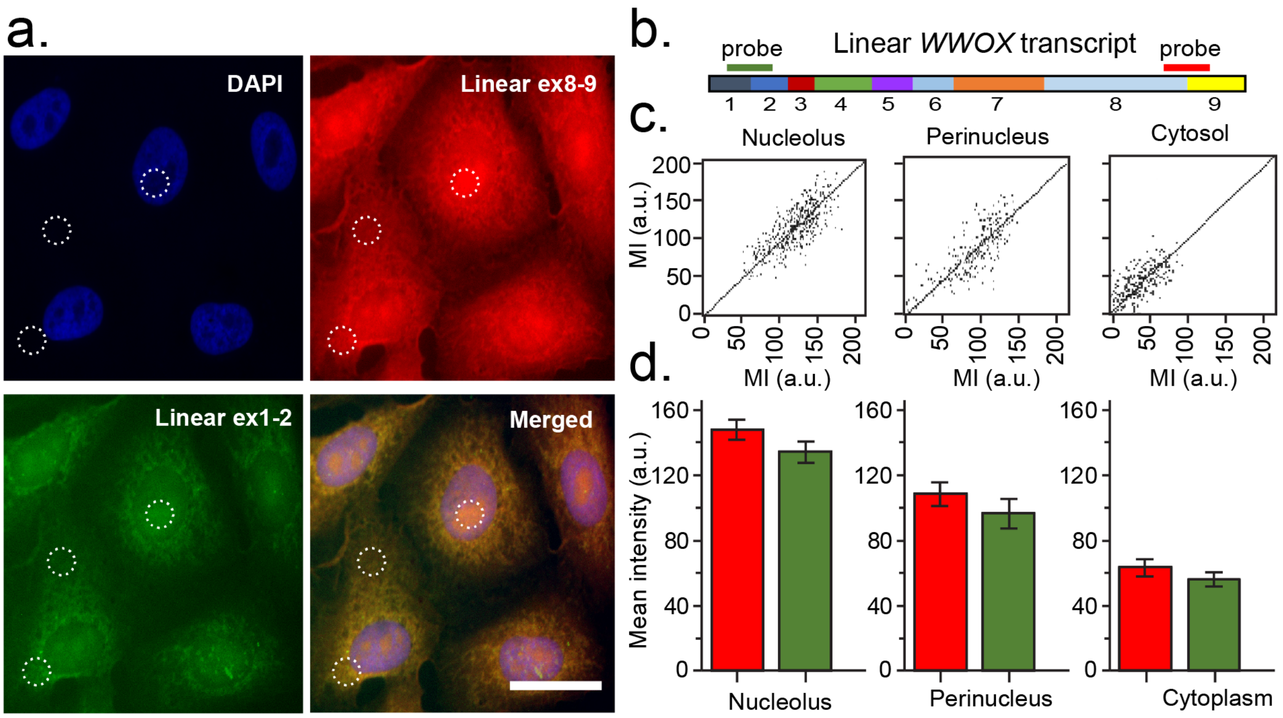

Supplementary Figure 4 related to Figure 3: Intracellular localisation of linear *WWOX* transcript control. (a) Representative epifluorescence micrographs of the linear *WWOX* mRNA transcript (referred to in the main text as *linWWOX*) identified with specific fluorescein dual end-labelled probes against the exon 1 - exon 2 boundary (ex1 - 2, green) and Quasar 570 dual end-labelled probes against the exon 8 - exon 9 boundary (ex8 - 9, red). Merged images (yellow) and DAPI stained nuclei (blue) shown. Scale bar 20 µm. **(b)** Schematic of the linear *WWOX* transcript and probe positions. Of note, exon 1 and 9 are absent from *KIRKOS-71* and *KIRKOS-73*. **(c)** Scatterplots of the co-localization of mean intensity (MI) signals (arbitrary units (a.u)) from Quasar 570 dual labelled probes (x – axis) or fluorescein dual labelled probes (y - axis) bound to the linear *WWOX* mRNA transcript. Histograms of the mean intensity of Quasar 570 dual labelled probes (red) or fluorescein dual labelled probes (green) bound to the linear *WWOX* mRNA transcript in the nucleolus, perinucleus or cytoplasm.

Supplementary Table 1 related to Figure 3: Intercellular co-localisation (R^2), percentage volume and number of co-localised pixels related to Quasar 570 dual end-labelled oligonucleotides binding to exon-exon junctions and Stellaris fluorescein labelled probes against the backbone of *WWOX* circRNAs *KIRKOS-71* and *KIRKOS-73* in the osteosarcoma cell line U2OS

U2OS	*KIRKOS-71*			*KIRKOS-73*		
	Nucleus	Perinucleus	Cytoplasm	Nucleus	Perinucleus	Cytoplasm
R^2 colocalisation	0.77 ± 0.026	0.82 ± 0.09	0.96 ± 0.03	0.72 ± 0.05	0.96 ± 0.07	0.86 ± 0.02
% volume	0.42	0.48	0.54	0.47	0.49	0.39
# colocalised pixels	226	258	290	258	293	286

Supplementary Table 2 related to Figure 2 and Figure 3: Sequences of primers and probes related to *KIRKOS-71* and *KIRKOS-73* utilised in this study

See Supplementary File 1
