# Supplementary material for: The circRNA interactome–innovative hallmarks of the intra- and extracellular radiation response
Source: Oncotarget. 2017 Jul 13;8(45):78397–409. doi: 10.18632/oncotarget.19228 (PMC5667970; doi:10.18632/oncotarget.19228)
Supplement: Supplementary file 2 [file oncotarget-08-78397-s002.doc]

| **Probe Name** | **Sequence** | **Label** |
| --- | --- | --- |
| ***KIRKOS-73* exon exon junction** | **gUUcaggUaccggagaagUa** | **Quasar 570 dual end-labelled** |
| ***KIRKOS-73* back bone** | **gccUcUUcaUgUgcUUgUgUgcaacgcagcaacUUUUgcUcUacccUggagUcUcaccaaagaUggccUggagaccaccUUUcaagUgaaUcaUcUggggcacUUcUaccUUgUccagcUccUccaggaUgUUUUgUgccgcUcagcUccUgcccgUgUcaUUgUggUcUccUcagagUcccaU** | **Stellaris FISH probes labelled with Fluorescein** |
| **Exon 1 and 2 boundary unique to linear *WWOX* transcript** | **CUGCCGACCCAAAUGAUGCGGUUAGUGUGGCUCCUCUUCUG** | **Fluorescein dual end-labelled probe** |
| **Exon 8 and 9 boundary unique to linear *WWOX* transcript** | **cuccggaaagugguucagguacguugucccucgacggugg** | **Quasar 570 dual end-labelled probe** |
| ***KIRKOS-73* left primer** | **AGTCCATGGCCTCTTCATGT** | **None** |
| ***KIRKOS-73* right primer** | **GTGCCCCAGATGATTCACTT** | **None** |
| ***KIRKOS-73* Taqman probe** | **AACGCAGCAACTTTTGCTCT** | **5’ FAM;**  **3’ TAMRA** |
| ***KIRKOS-71* exon exon junction** | **gagucuucuccucggugugacauggacuuggugaaaggcc** | **Quasar 570 dual end-labelled probe** |
| ***KIRKOS-71* backbone** | **AGUGGGAACAUCCAAAAACUGGAAAAAGAAAACGAGUGGCAGGAGAUUUGCCAUACGGAUGGGAACAAGAAACUGAUGAGAACGGACAAGUGUUUUUUGUUGACCAUAUAAAUAAAAGAACCACCUACUUGGACCCAAGACUGGCGUUUACUGUGGAUGAUAAUCCGACCAAGCCAACCACCCGGCAAAGAUACGACGGCAGCACCACUGCCAUGGAAAUUCUCCAGGGCCGGGAUUUCACUGGCAAAGUGGUUGUGGUCACUGGAGCUAAUUCAGGAAUAGGGUUCGAAACCGCCAAGUCUUUUGCCCUCCAUGGUGCACAUGUGAUCUUGGCCUGCAGGAACAUGGCAAGGGCGAGUGAAGCAGUGUCACGCAUUUUAGAAGAAUGGCAUAAAGCCAAGGUAGAAGCAAUGACCCUGGACCUCGCUCUGCUCCGUAGCGUGCAGCAUUUUGCUGAAGCAUUCAAGGCCAAGAAUGUGCCUCUUCAUGUGCUUGUGUGCAACGCAGCAACUUUUGCUCUACCCUGGAGUCUCACCAAAGAUGGCCUGGAGACCACCUUUCAAGUGAAUCAUCUGGGGCACUUCUACCUUGUCCAGCUCCUCCAGGAUGUUUUGUGCCGCUCAGCUCCUGCCCGUGUCAUUGUGGUCUCCUCAGAGUCCCAUCGAUUUACAGAUAUUAACGACUCCUUGGGAAAACUGGACUUCAGUCGCCUCUCUCCAACAAAAAACGACUAUUGGGCGAUGCUGGCUUAUAACAGGUCCAAGCUCUGCAACAUCCUCUUCUCCAACGAGCUGCACCGUCGCCUCUCCCCACGCGGGGUCACGUCGAACGCAGUGCAUCCUGGAAAUAUGAUGUACUCCAACAUUCAUCGCAGCUGGUGGGUGUACACACUGCUGUUUACCUUGGCGA** | **Stellaris FISH probes labelled with Fluorescein** |
| ***KIRKOS-71* left primer** | **GTCCATGTCACACCGAGGAG** | **None** |
| ***KIRKOS-71* right primer** | **CATCCACAGTAACGCCAGT** | **None** |
| ***KIRKOS-71* Taqman probe** | **CGGATGGGAACAAGAAACTG** | **5’ FAM;**  **3’ TAMRA** |

**Table S2 related to Fig.2 and Fig.3.** Sequences of primers and probes related to *KIRKOS-71* and *KIRKOS-73* utilised in this study.
